# Fabrication of Metal–Organic Framework-Mediated Heterogeneous Photocatalyst Using Sludge Generated in the Classical Fenton Process

**DOI:** 10.3390/nano15141069

**Published:** 2025-07-10

**Authors:** Xiang-Yu Wang, Xu Liu, Wu Kuang, Hong-Bin Xiong

**Affiliations:** 1 School of Resources and Environmental Engineering, Hefei University of Technology, Hefei 230009, China; wangxiangyu1618@163.com; 2 Anhui Provincial Academy of Eco-Environmental Science Research, Hefei 230061, China; kuangwu1972@126.com; 3 AnHui Environmental Science and Technology Group Co., Ltd., Hefei 230088, China

**Keywords:** Fenton wastes, metal–organic framework, heterogeneous photo-Fenton catalyst, methylene blue

## Abstract

The sludge produced by the Fenton process contains mixed-valence iron particulates (hereafter called Fenton wastes). Using a solvothermal method, we fabricated a new heterogeneous photo-Fenton catalyst using Fenton wastes and metal–organic frameworks (MOFs). Nanoporous metal carboxylate (MIL-88) MOF impregnated with Fenton waste was functionalized using 2,5-dihydroxyterephthalic acid (x-HO-MIL-88-C, x, concentration of the 2,5-dihydroxyterephthalic acid). The efficiency of x-HO-MIL-88-C was examined under visible light radiation using methylene blue (MB) as an index pollutant. We observed the best catalytic performance for MB degradation by 4-HO-MIL-88-C. In the photo-Fenton process, the simultaneous presence of singlet oxygen, superoxide, and hydroxyl radicals is confirmed by free radical quenching and electron spin resonance spectral data. These free radicals associate with holes in the non-selective degradation of MB. The 4-HO-MIL-88-C catalyst shows good stability and reusability, maintaining over 80% efficiency at the end of five consecutive cycles. This work opens up a new path for recycling Fenton wastes into usable products.

## 1. Introduction

Rapid industrialization has increased the discharge of recalcitrant organic pollutants such as dyeing wastewater into the environment, posing a serious threat to ecosystems. The conventional treatments such as precipitation [1], membrane filtration [2,3], and biological oxidation [4] are not efficient in removing recalcitrant organic pollutants. The ozone oxidation of organic pollutants shows some promise [5,6]; however, the cost of the technology is high, and sometimes partial oxidation by O_3_ occurs, inducing secondary pollution. The hydroxyl radical (standard redox potential 2.8 V, oxidation rate ~10^9^ M^−1^s^−1^) is hyper-reactive [7], and, in most cases, non-selectively destructs organic pollutants into harmless products. The conventional Fenton process produces OH efficiently in the presence of Fe^2+^ and H_2_O_2_ at acidic pH. However, this process suffers from excess sludge generation, initial chemical cost, and extreme solution acidity [8]. Different variants of the Fenton process have been introduced, such as heterogeneous Fenton [9], fluidized Fenton [10], electrochemical Fenton [11], bio-electrochemical Fenton [12], etc. However, as discussed elsewhere, these variants have inherent deficiencies, which results in the conventional Fenton process being operational even today to destroy organic pollutants.

Metal–organic frameworks (MOFs), a new type of semiconductor photocatalyst, are widely used in biosensing [13], drug delivery [14], adsorption [15], antibacterial [16], and advanced oxidation [17] due to their good chemical stability and adjustable bandgap. A MOF is synthesized by coordination bonds between metal ions or clusters and organic ligands. It has high porosity, a wide specific surface, and adjustable pores. Studies have confirmed that the role of ligand–metal charge transfer (LMCT) could promote charge separation, so a MOF can provide a large number of reactive sites, showing great potential in photocatalysis. Among the many types of MOFs, MIL-88(Fe) has attracted wide attention due to its high specific surface area and good thermal stability. However, the original MIL-88 exhibits poor photocatalytic performance due to its limited light-induced charge separation and light utilization capabilities. We used two methods to improve the photocatalytic activity of MIL-88: (i) Introduce conductors or semiconductors into MIL-88 to form a heterojunction, such as MIL-88/g-C_3_N_4_ [18], MIL-88/Bi_2_WO_6_ [19], etc., to enhance the visible light absorption of MIL-88 and improve the photocatalytic performance of the composite materials; (ii) Introduce –NH_2_, –OH, and other functional groups to the organic ligand of MIL-88 to improve the photocatalytic performance. The functionalized NH_2_-MIL-88/HO-MIL-88 shows a wider range of visible light absorption, enhancing the separation of charge carriers, improving the electron transport from the organic linker to the Fe-O cluster, and enhancing the excitation of the Fe-O cluster. However, the prepared MIL-88 crystals are prone to agglomeration, decreasing the catalytic performance. The sludge after the Fenton reaction is rich in iron and has a low utilization rate. Using this sludge as a raw material to prepare MOF can provide metal ions for the synthesized catalyst and serve as a carrier to fix the photocatalyst on the sludge surface. The resource utilization of sludge is realized, and the agglomeration effect of photocatalysis is avoided.

This work proposed a simple one-step solvothermal synthesis method to fabricate the HO-MIL-88/C catalyst under visible light irradiation using the Fenton wastes. The density of hydroxyl groups of the MIL-88/C was modified by 2,5-dihydroxyterephthalic acid. Scanning electron microscopy (SEM), transmission electron microscopy (TEM), X-ray diffraction (XRD), Fourier transform infrared spectrometry (FT-IR), X-ray photoelectron spectrometry (XPS), and electrochemical impedance spectroscopy (EIS) were used to characterize the physical and chemical properties of the catalyst. Methylene blue was used as an index pollutant to optimize experiment conditions of the HO-MIL-88/C catalyst, e.g., H_2_O_2_ concentration, pH, and initial pollutant concentration. Further, the catalyst’s mechanism of methylene blue degradation was proposed based on free radical scavenging experiments and electron spin resonance (ESR) measurements.

## 2. Experimental

### 2.1. Chemicals

All chemicals were analytical grade and used without further purification. 2,5-dihydroxyterephthalic acid was from Sinopharm Chemical Reagent Co., Ltd. (Shanghai, China). Hydrochloric acid (HCl), sodium hydroxide (NaOH), N,N′-dimethylformamide (DMF), hydrogen peroxide (H_2_O_2_), methylene blue (MB), sodium sulfate (Na_2_SO_4_), silver nitrate (AgNO_3_), tert-butanol (TBA), ethylenediaminetetraacetic acid disodium salt (EDTA-2Na), 1,4-benzoquinone (BQ), and furfuryl alcohol (FFA) were obtained from Shanghai Macleans Chemical Co., Ltd. (Shanghai, China). 2,2,6,6-Tetramethyl-1-piperinedinyloxy (TEMPO) and 5,5-dimethyl-1-pyrroline N-oxide (C_6_H_11_NO, DMPO) were bought from Sigma-Aldrich (St. Louis, MO, USA). Deionized water was used in all sample preparations. The sludge used in the experiment was collected from the coagulation sedimentation tank of an internal wastewater treatment plant at a dye chemical manufacturing plant in Anqing City, with its elemental composition and content shown in Table S1.

### 2.2. Preparation of HO-MIL-88/C Composites

The composites were synthesized by a one-step solvothermal method with a previously reported procedure [14]. First, 20 g of the Fenton wastes was dried in an oven at 80 °C for 12 h and then ground into powder form. Secondly, 1.00 g of the sludge powder was taken separately with 80 mL of DMF solution and dispersed under stirring for 4 h. 2,5-Dihydroxyterephthalic acid was added to the aforementioned solutions at concentrations of 2 mM, 3 mM, 4 mM, and 5 mM, respectively, followed by thorough mixing. Thirdly, the mixed solutions were transferred to an autoclave lined with polytetrafluoroethylene, heated at 120 °C for 24 h, cooled to ambient atmospheric temperature, and washed with deionized water 5 times to remove residual impurities. The purified catalyst was dried in an oven at 60 °C for 8 h and then ground into powder. The powders obtained were named x-HO-MIL-88 (x = 2~5).

### 2.3. Characterization

The crystal structures of x-HO-MIL-88/C composites were determined in the range of 2θ = 5~80° by fixed-target X-ray diffraction (X-Pert PRO MPD PANalytical, Almelo, The Netherlands ). The morphology, microstructure, and elemental composition of the material were determined by a cold field emission scanning electron (SEM; SU 8020 Hitachi, Tokyo, Japan), JEM-2100F transmission electron microscopic methods (HRTEM; JEM 2100F JEOL, Tokyo, Japan), and energy-dispersive X-ray spectrometer (EDS, Carl Zeiss AG, Oberkochen, Germany). The electron transfer processes at the solid–solution interface were examined by electrochemical impedance spectroscopy at a frequency range of 100,000~1 Hz (ChenHua Instruments, Shanghai, China). The functional groups and elemental states of the x-HO-MIL-88/C were examined by a Fourier infrared spectrometer (FT-IR, Thermo Nicolet Co., Waltham, MA, USA) and X-ray photoelectron spectrometer (XPS, PHI 5000 Versa Probe instrument, PHI, Chanhassen, MN, USA).

### 2.4. Photo-Fenton Experiments

The efficiency of the x-HO-MIL-88/C photocatalysts was examined using methylene blue as an index pollutant. In a typical methylene blue degradation experiment, a 20 mg/L catalyst was used. After regulating the desired pH using 0.1 mol/L HCl or 0.1 mol/L NaOH (pH meter HQ30d; Hach, USA), the reaction was commenced with 50 mL methylene blue for 10 min in the dark to reach adsorption equilibrium. Afterward, the reactor was irradiated with a 500 W Xe lamp (equipped with a 420 nm cut-off filter, light intensity 200 mW/cm^2^). In 10 min intervals, sample aliquots were filtered (0.45 μm) into vials to determine residual methylene blue concentration by UV–vis spectroscopy at λ = 655 nm (UV 2600, Shimadzu, Tokyo, Japan).

### 2.5. Solution Free Radicals’ Detection

The presence of •OH, •O_2_^−^_,_ and ^1^O_2_ radicals in the reaction solution was determined by electron spin resonance spectroscopy (JES-FA 200; Jeol, Tokyo, Japan) after capturing them with 5,5-dimethyl-1-pyrroline N-oxide (DMPO) and 2,2,6,6-Tetramethyl-1-piperidinyloxy (TEMPO).

## 3. Results and Discussion

### 3.1. Structural and Morphological Characterizations

**Figure 1:**
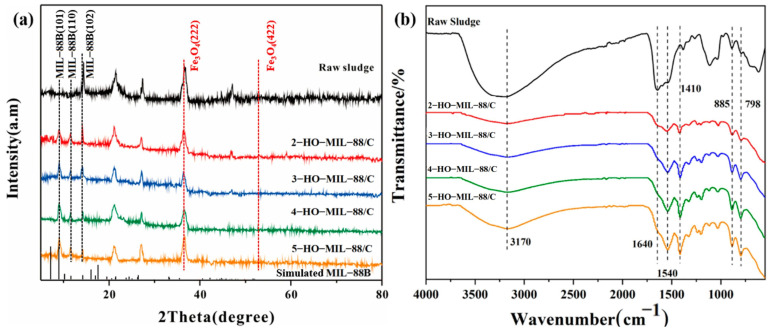
(**a**) XRD patterns; (**b**) FT-IR spectra of synthesized catalysts.

Figure 1a shows the XRD diffractograms of x-HO-MIL88/C. Compared to the raw Fenton wastes (black color XRD), in x-HO-MIL-88/C, two new peaks at 2θ = 9.1° and 12.2°, which correspond to the (101) and (110) planes of MIL-88B, appeared [20]. The peaks at 2θ = 22° and 27° may correspond to aniline derivatives from carbon impurities in the substrate and minor graphitic carbon present in the catalyst, respectively [20]. The XRD peaks are shown at 2θ = 36.1 and 53.1 in all samples corresponding to (222) and (422) crystal planes of Fe_3_O_4_ (JCPDS#79-0419) [21].

As shown in Figure 1b, the degree of MIL-88/C functionalization by 2,5-dihydroxyterephthalic acid was examined by IR spectroscopy. In all samples, viz. Fenton wastes and x-HO-MIL-88/C composites, typical broadband around 3700–2600 cm^−1^ centered at 1640 cm^−1^ to -OH groups are discerned. The latter IR band (1640 cm^−1^) confirms the presence of x-HO-MIL-88/C surface-adhered -OH groups [22,23]. The intensity of this peak has increased with the concentration of 2,5-dihydroxyterephthalic acid used for x-HO-MIL-88/C fabrication. The peaks at 1540 cm^−1^ and 1410 cm^−1^ are ascribed to the asymmetric and symmetric stretching vibrations of -COO- [24,25]. The distinct peaks at 885 cm^−1^ and 798 cm^−1^ are due to the Fe-O bending vibrations [26].

**Figure 2:**
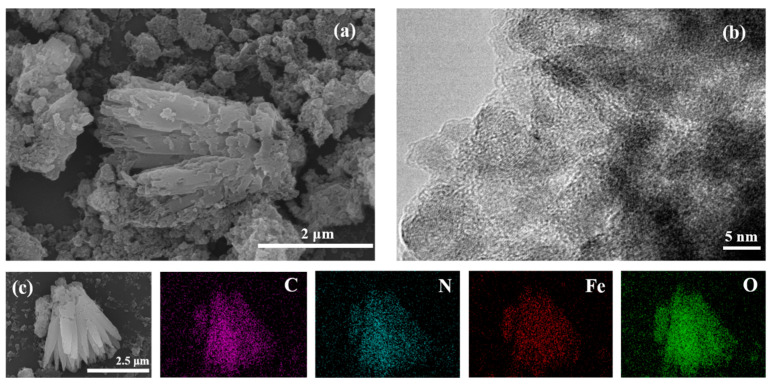
Characterization of 4-HO-MIL-88/C. (**a**) SEM image; (**b**) TEM image; (**c**) elemental mapping images.

The morphology and microstructures of the x-HO-MIL-88/C composites were recorded by SEM and TEM microscopic methods, as shown in Figure 2. The 4-HO-MIL-88/C is different from a typical MIL-88 MOF for the following points (Figure 2a). Figure 2a shows that 4-HO-MIL-88/C exhibits a lamellar stacking structure, which tightly adheres to the surface of the Fenton waste, with a particle size of about 200–300 nm, which is significantly smaller than that of the conventional MIL-88 (400 nm octahedron), and is more homogeneously distributed. In Figure 2b, a typical MIL-88 is shown, with an octahedral-shape crystal of around 400 nm dimensions; however, the 4-HO-MIL-88/C crystal forms a layered structure and grows in situ on the surface of the Fenton wastes. The 4-HO-MIL-88/C crystal grows in clusters. The FETEM image of the 4-HO-MIL-88/C is shown in Figure 2b. Figure 2c displays the EDS mapping image of the 4-HO-MIL-88/C. The C, N, O, and Fe elements are evenly distributed in the catalyst, indicating that it was successfully synthesized.

### 3.2. Photo-Fenton Catalytic Activity Analysis

**Figure 3:**
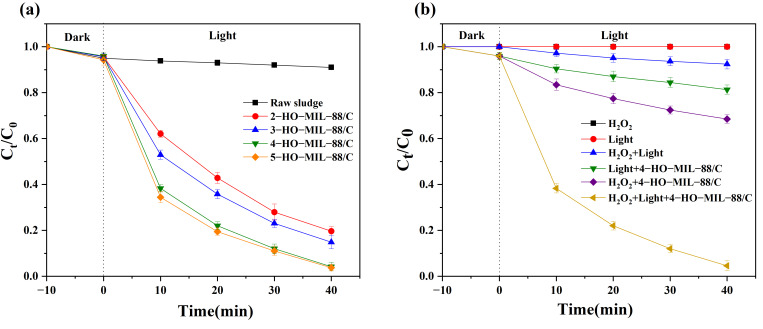
(**a**) The degradation of MB by different catalysts; (**b**) The effect of different reaction systems on the MB degradation (pH = 3, [H_2_O_2_] = 2 mM, [MB] = 20 mg/L, catalyst = 200 mg/L).

In the absence of visible light, the methylene blue adsorption on x-HO-MIL-88/C and Fenton wastes was examined in Figure S1 (Supplementary Materials). An optimal methylene blue adsorption is reached within the first ten minutes (therefore, ten minutes of reaction lapse time was allowed before irradiation). The methylene blue degradation efficiency by x-HO-MIL-88/C was optimized as a function of 2,5-dihydroxyterephthalic acid loading, methylene blue, and protons concentration. The methylene blue degradation by the photocatalyst increases when 4-HO-MIL/C is used (4 mM 2,5-dihydroxyterephthalic acid was used in the catalyst fabrication) in Figure 3a. Therefore, the 4-HO-MIL/C composite was used in other experiments.

As shown in Figure 3b, MB was not degraded with visible light irradiation or H_2_O_2_ addition. However, when the MB/[H_2_O_2_] system is irradiated by visible light, only 7.6% methylene blue degraded within 40 min. When the MB/4-HO-MIL-88/C system was irradiated, only 18.7% MB was removed from the solution. However, when [H_2_O_2_] is added to MB/4-HO-MIL-88/C, the degradation rate increases up to 31.5% in the dark, which is ascribed to a homogeneous Fenton reaction. In the presence of visible light, due to synergistic effects, the number of holes and electrons generation is increased, which results in increased free radicals’ production.

**Figure 4:**
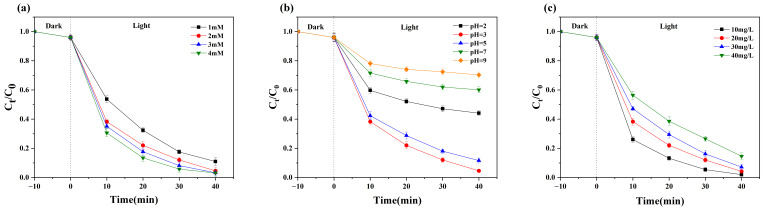
The effect of various parameters on the MB degradation in 4-HO-MIL-88/C-catalyzed photo-Fenton system: (**a**) (pH = 3, [MB] = 20 mg/L, catalyst = 200 mg/L); (**b**) ([H_2_O_2_] = 2 mM, [MB] = 20 mg/L, catalyst = 200 mg/L); (**c**) (pH = 3, [H_2_O_2_] = 2 mM, catalyst = 200 mg/L).

Variations of [H_2_O_2_] on MB degradation by the 4-HO-MIC-88/C composite are shown in Figure 4a. When [H_2_O_2_] concentration reaches beyond 2 mM, the MB degradation shows a monotonous behavior. In subsequent experiments, the [H_2_O_2_] concentration is always kept at 2 mM.

The solution pH affects the •OH production rate, which is crucial for MB degradation. As shown in Figure 4b, when the pH increased from ~2.00 to ~3.00, the methylene blue degradation increased by 40.2% within 40 min. When solution pH < ~3.00, •OH radicals readily scavenged H^+^_,_ increasing the catalyst’s corrosion rate or decreasing its stability. When solution pH > 3.00, MB degradation has declined from 95.8% (pH ~3.00) to 30.7% (pH ~9.00), which can be ascribed to the following reasons: (1) the oxidation–reduction potential of free radicals decreases with the pH increase; (2) the stability of H_2_O_2_ reduced promoting its self-decomposition. Therefore, the optimal pH for the degradation of MB kept around 3.00.

The initial loading of MB affects the photocatalyst’s efficiency (Figure 4c). When initial [MB] < 20 mg/L, MB degradation efficiency exceeds 95% (Figure 4c). However, when the MB concentration increases beyond 20 mg/L, its degradation decreases the number of active sites in the catalyst.

### 3.3. Active Species Detection

**Figure 5:**
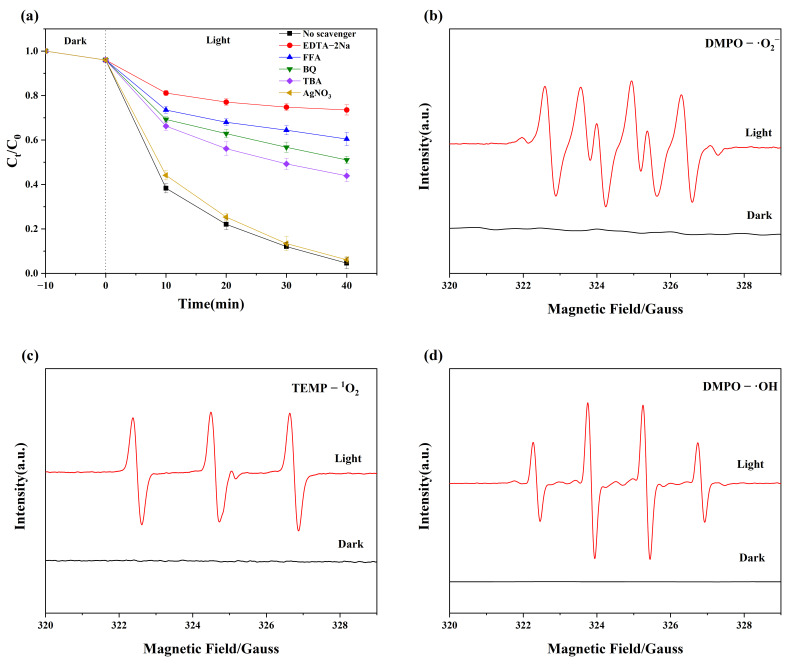
(**a**) Effect of different reactive species scavengers; (**b**) DMPO-•O_2_^−^ adducts; (**c**) TEMPO-^1^O_2_ adducts; (**d**) DMPO-•OH.

The effect of free radicals in the 4-OH-MIF-88/C photocatalyst was also examined using free radical quenchers and electron spin resonance (ESR) spectroscopic measurements. The results are shown in Figure 5a–d. Tert-butanol (TBA, 100 mM), p-benzoquinone (BQ, 4 mM), EDTA-Na (4 mM), AgNO_3_ (10 mM), or FFA (10 mM) were used in quenching •OH, •O_2_^−^, h^+^, e^−^, or ^1^O_2_, radicals, respectively [27]. As noted elsewhere [28], AgNO_3_ readily scavenges free electrons. As shown in Figure 5a, the addition of AgNO_3_ did not affect the methylene blue degradation rate. However, EDTA-Na, FFA, BQ, and TBA scavenge •OH, •O_2_^−^, h^+^, and ^1^O_2_, respectively. As shown in Figure 5a, these radicals (e.g., •OH, •O_2_^−^, h^+^, and ^1^O_2_) degrade MB in various proportions. Accordingly, when EDTA-Na, FFA, BQ, or TBA is present in the reactor, the efficiency of MB degradation decreased from 95.8% to 26.5%, 39.6%, 48.9%, and 56.1%, respectively. Therefore, the effect of the free radicals on methylene blue degradation can be ordered h^+^ > ^1^O_2_ > •O_2_^−^ > •OH > e^−^.

We also observed the presence of •OH, •O_2_^−^, and ^1^O_2_ radicals in the photoreactor by ESR measurements using DMPO/TEMPO trapping reagents within the first 5 min after the commencement of the reaction. The ESR measurements were carried out for samples prepared under dark and light irradiation (Figure 5b–d). Figure 5c shows that the ^1^O_2_ signal is absent in the dark; however, a distinct TEMPO-^1^O_2_ signal appeared under 500 W xenon lamp irradiation, confirming the presence of ^1^O_2_. Similarly, as shown in Figure 5c,d, we could not observe signals corresponding to •O_2_^−^ and •OH in the dark, but they appeared upon light irradiation. In agreement with quenching experiments (Figure 5a), these results confirmed the reactor’s concurrent presence of •OH, •O_2_^−^_,_ and ^1^O_2_ radicals.

### 3.4. Mechanism of Photo-Fenton Catalytic Degradation

Electrochemical impendence spectroscopic (EIS) measurements were made with the x-HO-MIL-88/C modified glassy carbon electrode using 5 mM Fe (CN_6_^3−/4−^) in 0.1 M KCl to probe the electrons’ transfer mechanism at the solid and solution interface. In the EIS plots, the radius of the semi-circle (R_et_) and the linear sections characterize electrons transfer and ion diffusion, respectively [29]. As shown in Figure S2, the electron transfer rate increased with the number of organic ligands (e.g., 4 mM 2,5-dihydroxyterephthalic acid) in the x-HO-MIL-88/C composite, reaching an optimum at 4:1 organic ligand to metal atoms ratio [29]. The presence of organic ligands increases the number of oxygen vacancies in MOF. Table S2 shows the circuit simulation parameters of EIS data, and the resistances of 2-HO-MIL-88/C, 3-HO-MIL-88/C, 4-HO-MIL-88/C, and 5-HO-MIL-88/C are 123.2 Ω, 108.1 Ω, 97.8 Ω, and 115.3 Ω, respectively (inset of Figure S2). Accordingly, when the ligand concentration in the x-HO-MIL-88/C increases further, it inhibits the transfer of electrode/ solution interfacial electrons.

**Figure 6:**
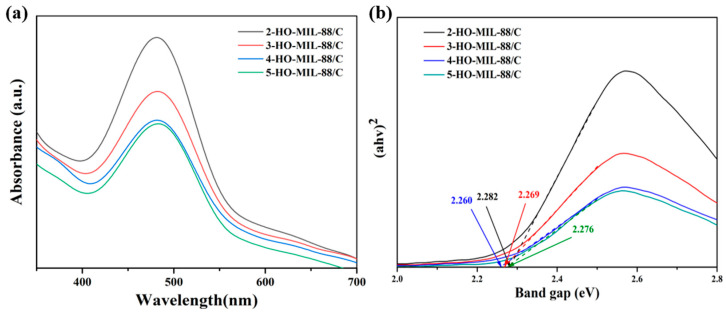
(**a**) UV-DRS spectra of four catalysts; (**b**) Facet-dependent bandgap variations in four heterogeneous catalysts.

The optical properties of the x-HO-MIL-88/C catalysts were also examined by UV–vis diffuse reflectance spectroscopy (DRS), as shown in Figure 6. All samples show distinct absorption edges. the bandgap energy (E_g_) is estimated by a T_auc_ plot using the Kubelka–Munk function:

(1) αhv = A(hv − E_g_)^n/2^

where α, A, h, and v stand for absorption coefficient, the proportionality constant, Planck constant, and light frequency, respectively. The parameter n for MIL-88 with a direct transition feature is 1. The bandgap of 2-HO-MIL-88/C, 3-HO-MIL-88/C, 4-HO-MIL-88/C, and 5-HO-MIL-88/C were 2.282 eV, 2.269 eV, 2.260 eV, and 2.276 eV, respectively (the inset of Figure 6). Among these, 4-HO-MIL-88/C exhibits the lowest band gap value. The valence band potential was measured by XPS spectra, as shown in Figure S3; the VB value of 4-HO-MIL-88/C was 1.71 eV. Accordingly, the conduction band potential of 4-HO-MIL-88/C was estimated as −0.55 eV.

(2) CB = VB − E_g_

where CB represents the conduction band, an energy region where electrons can move freely in the semiconductor; VB represents the valence band, an energy region where electrons are bound around atoms and can transition to the conduction band after gaining sufficient energy; E_g_ represents the band gap energy, the energy difference between the conduction band and valence band, which determines the light absorption and photocatalytic activity of the semiconductor.

**Figure 7:**
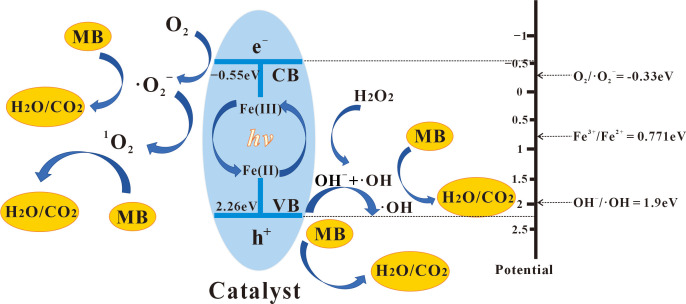
Schematic illustration of proposed photocatalytic mechanism.

Accordingly, we proposed a possible mechanism for the methylene blue degradation by 4-HO-MIL-88/C catalyst as in Figure 7. The 4-HO-MIL-88/C catalyst photo-generated electrons and holes after irradiation with visible light, and the exciting ligands transferred photoelectrons to the metal cluster. Due to negative CB potential (−0.55 eV), the excited electrons in 4-HO-MIL-88/C reduced dissolved O_2_ to •O_2_^−^ [29]; then, it (•O_2_^−^) combined with h^+^-generating ^1^O_2_. Free e^−^ reacted with H_2_O_2_ to generate •OH and OH^−^_,_ and it was continuously oxidized to •OH by h^+^ [29]. Therefore, h^+^, •OH, •O_2_^−^_,_ and ^1^O_2_ play an important role in photocatalytic methylene blue degradation. It is worth noting that this study did not track the mineralization pathway of MB, and the degradation products might be small-molecule organic substances rather than complete inorganic ones. Subsequently, the reaction mechanism needs to be further clarified by combining mass spectrometry and TOC analysis.

The reaction process can be described as follows (Equations (3)–(13)):

(3) 4-HO-MIL-88/C + *hv* → e^−^ + h^+^

(4) Fe^3+^ + e^−^ → Fe^2+^

(5) Fe^2+^ + H_2_O_2_
→ Fe(OH)^2+^ + •OH

(6) Fe(OH)^2+^ → Fe^3+^ + OH^−^

(7) O_2_ + e^−^ → •O_2_^−^

(8) H_2_O_2_ + e^−^ → •OH + OH^−^

(9) OH^−^ + h^+^ → •OH + e^−^

(10) H_2_O_2_ → H^+^ + HO_2_^−^

(11) H_2_O_2_ + HO_2_^−^ → ^1^O_2_ + H_2_O + OH^−^

(12) 2•O_2_^−^ + 2 H_2_O → ^1^O_2_ + H_2_O_2_ + 2 OH^−^

(13) h^+^/^1^O_2_/•O_2_^−^/•OH + MB → small molecule organic substances

### 3.5. Stability and Reusability of the Catalyst

**Figure 8:**
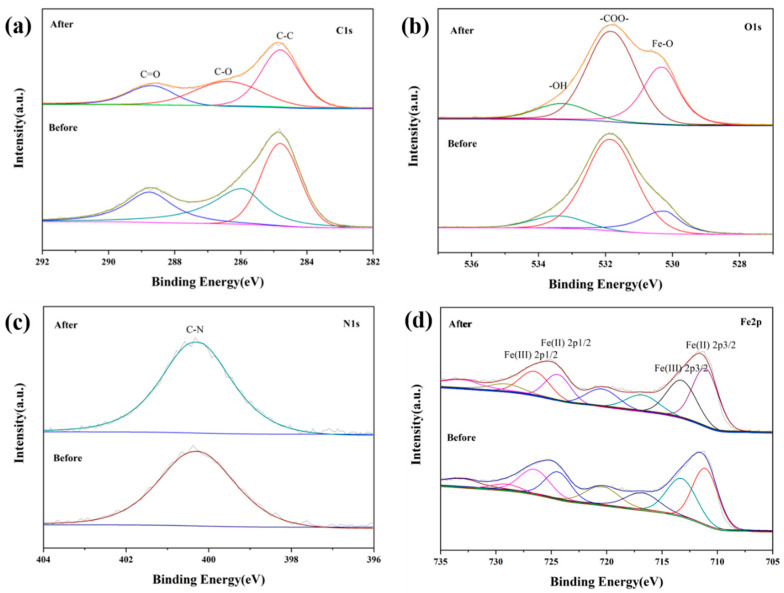
XPS spectra of (**a**) C 1s, (**b**) O 1s, (**c**) N 1s, and (**d**) Fe 2p for the 4-HO-MIL-88/C composite before and after photo-Fenton reactions.

The elemental composition and the chemical states of the 4-HO-MIL-88/C catalysts (e.g., virgin and spent) were examined by X-ray photon spectroscopy (XPS), as shown in Figure 8. The 1s C XPS peaks at 284.8 eV, 285.9 eV, and 288.7 eV for the virgin 4-HO-MIL-88/C correspond to C–C, C–O, and C=O, respectively (Figure 8a). As methylene blue degradation commences, the C-C peak shifts left by 0.4 eV while the other C1s peaks correspond to C-O and C=O remained unchanged (Figure 8b). Further, the O1s peak at 530.2 eV of the Fe-O bond association shows a marked variation as the reaction proceeds. However, the O1s at 533.3 eV corresponds to O-H and shows no variation confirming the robustness of the catalyst’s activity for repeated use. The N1s peak at 400.6 eV indicates the presence of organic C–N in the sludge; however, its peak intensity did not vary between virgin and spent catalyst. Four peaks at 709.9 eV, 712.1 eV, 723.3 eV, and 725.9 eV were assigned to Fe (II) 2p3/2, Fe (III) 2p3/2, and Fe (II) 2p1/2, Fe(III) 2p1/2, respectively, and the four peaks at 715.8 eV, 719.4 eV, 730.0 eV, and 733.4 eV can be attributed to Fe(II) 2p3/2, Fe(III) 2p3/2, Fe(II) 2p1/2, and Fe(III) 2p1/2 (satellite peak). The ubiquitous presence of Fe (III)/Fe (II) mixed-valence states in the catalyst required for the photo-Fenton process is thus confirmed.

**Figure 9:**
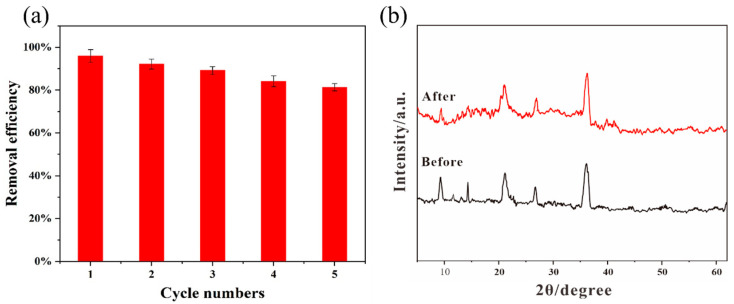
(**a**) Removal efficiency of MB (Experimental conditions: pH = 3, [MB] = 20 mg/L, catalyst = 200 mg/L, [H_2_O_2_] = 2 mM). (**b**) XRD patterns before and after reaction.

The robustness of the 4-HO-MIL-88/C catalyst is important in practical applications. To evaluate the stability of the catalyst, five consecutive cyclic experiments were conducted under the following initial conditions: the concentration of 4-HO-MIL-88/C catalyst was 0.20 g/L, [H_2_O_2_] was 2 mM, [methylene blue (MB)] was 20 mg/L, and the solution pH was 3.00. At the end of each methylene blue degradation cycle, the spent catalyst is washed and dried at 80 °C before the next step. As shown in Figure 9a, the methylene blue removal efficiency diminished slightly from 95.9% to 81.3% after repeated catalyst use. The reduction of active sites on the catalyst covered with Fe (III) colloids is attributed to a slightly diminished performance. By comparing the XRD patterns of 4-HO-MIL-88/C before and after the reaction (Figure 9b), it can be seen that the material exhibits no significant phase change, further confirming its strong stability. However, our results indicate a good reusability and stability of the 4-HO-MIL-88/C catalyst.

## 4. Conclusions

We fabricated a novel photo-Fenton catalyst, viz. 4-HO-MIF-88/C, using the sludge generated from the conventional Fenton process to destroy MB, showing greater than 96% efficiency via non-selective degradation under visible light irradiation. Hydroxyl radicals facilitate photoelectrons and hole separation, enhancing the catalyst activity. Electron spin resonance spectral data confirm the presence of free radicals such as •OH, •O_2_^−^, h^+^, e^−^, and ^1^O_2_ in the reaction system. The 4-HO-MIL-88/C catalyst is robust and can repeatedly be used in multiple cycles to destroy organic pollutants in wastewater.

## Data Availability

The original contributions presented in this study are included in the article/Supplementary Material. Further inquiries can be directed to the corresponding author(s).

## Supplementary Materials

The following supporting information can be downloaded at: https://www.mdpi.com/article/10.3390/nano15141069/s1, Figure S1. Adsorption capacities of raw sludge and prepared catalysts. Figure S2. EIS Nyquist plots of prepared catalysts. Figure S3. VB XPS spectra of 4-HO-MIL-88/C. Table S1. The chemical properties of sludge. Table S2. Parameters of EIS equivalent circuit components.
