# Supplementary material for: Fabrication of Metal–Organic Framework-Mediated Heterogeneous Photocatalyst Using Sludge Generated in the Classical Fenton Process
Source: Nanomaterials (Basel). 2025 Jul 10;15(14):1069. doi: 10.3390/nano15141069 (PMC12300062; doi:10.3390/nano15141069)
Supplement: Supplementary file 1 [file nanomaterials-15-01069-s001.zip › nanomaterials-3619565-supplementary.pdf]

Fabrication of Metal-Organic Frameworks mediated heterogeneous photocatalyst using sludge generated in classical Fenton process

Xiang-Yu Wang [1, 2] Xu Liu [1, 3]*, Wu Kuang [2] and Hong-Bin Xiong [1]*

1 School of Resources and Environmental Engineering, Hefei University of Technology, Hefei 230009, P. R. China

2 Anhui Provincial Academy of Eco-Environmental Science Research, Hefei 230061, P R China

3 AnHui Environmental Science and Technology Group Co., Ltd., Hefei, 230088, P. R. China

* Correspondence: Xu Liu: 2025870001@hfut.edu.cn;

Hong-Bin Xiong:xhb6324@sina.com

Table of contents

Figure S1. Adsorption capacities of raw sludge and prepared catalysts.

Figure S2. EIS Nyquist plots of prepared catalysts.

Figure S3. VB XPS spectra of 4-HO-MIL-88/C.

Table S1. The chemical properties of sludge.

Table S2. Parameters of EIS equivalent circuit components

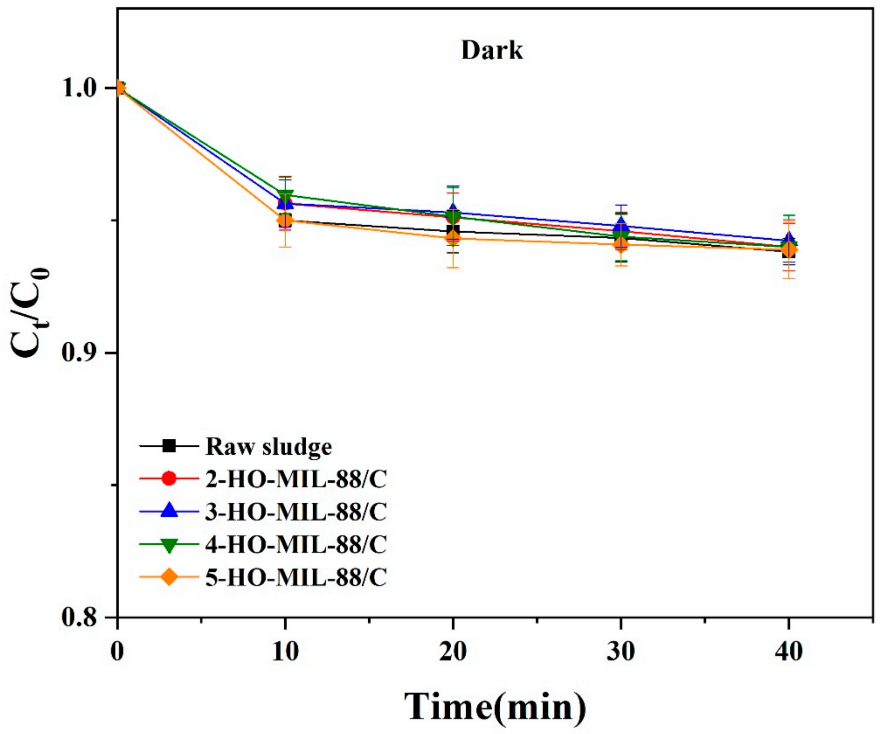

Figure S1. Adsorption capacities of raw sludge and prepared catalysts. (pH = 3, [MB] = 20 mg/L, catalyst = 200 mg/L)

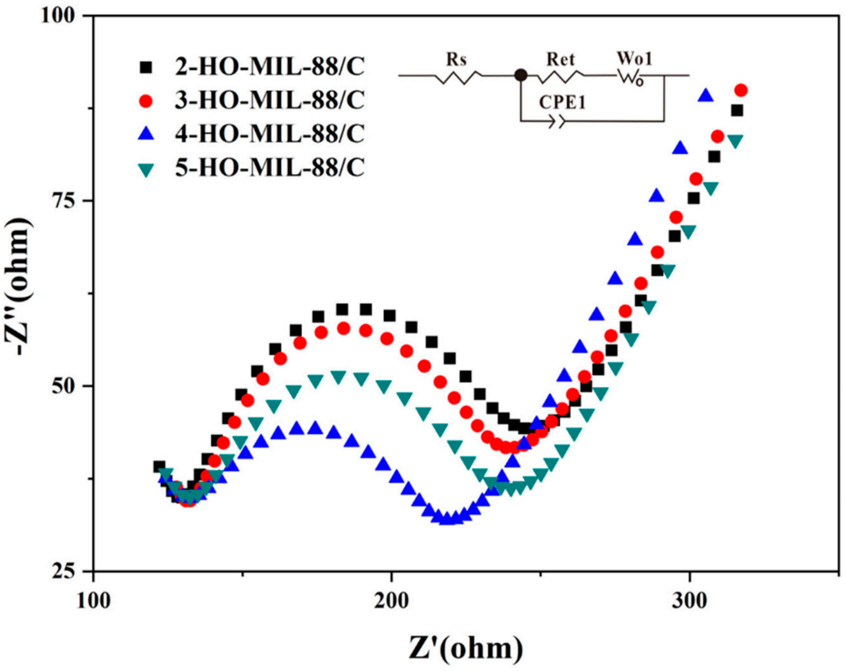

Figure S2. EIS Nyquist plots of prepared catalysts.

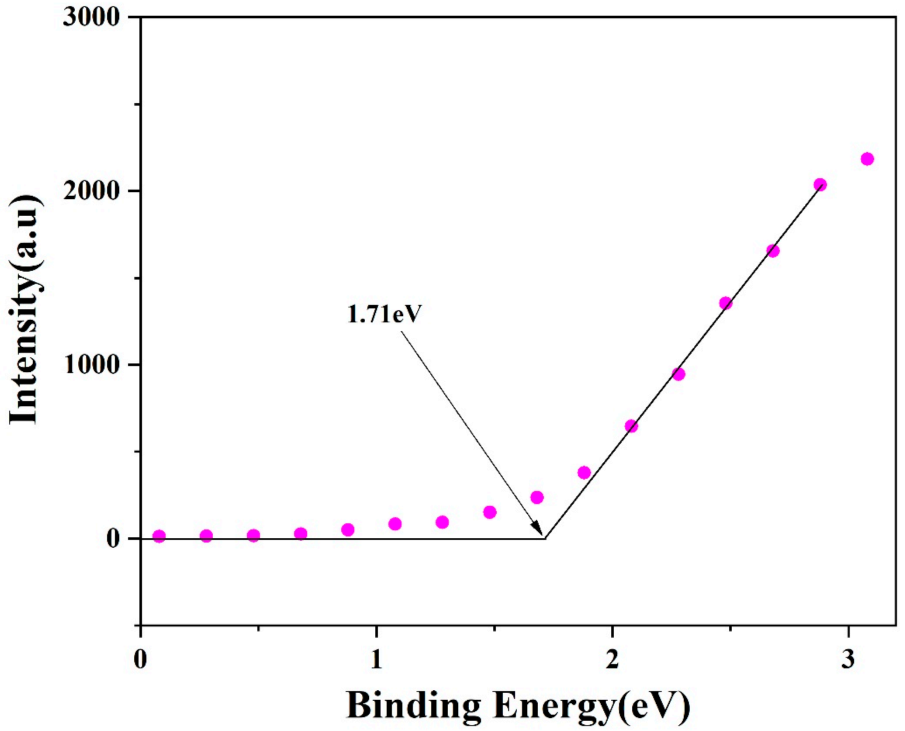

Figure S3. VB XPS spectra of 4-HO-MIL-88/C.

Table S1. The chemical properties of sludge.

Chemical elements	content	Chemical elements	content
Fe	68.57%	Si	0.47%
O	8.64%	Na	0.23%
S	5.23%	P	0.21%
C	5.08%	Mg	0.13%
N	3.64%	Cr	0.09%
Br	3.40%	Zn	0.08%
Cl	1.62%	Ni	0.06%
Ti	1.26%	Cu	0.03%
Mn	0.63%	K	0.03%
Ca	0.60%		

Table S2. Parameters of EIS equivalent circuit components

Electrodes / Parameter	2-HO-MIL-88/C	3-HO-MIL-88/C	4-HO-MIL-88/C	5-HO-MIL-88/C
Rs	117.1	113.3	110.5	116.5
Ret	123.2	108.1	97.8	115.3
CPE-T	1.4385×10^{-6}	1.0103×10^{-6}	1.6602×10^{-6}	1.5584×10^{-6}
CPE-P	0.8638	0.8671	0.8228	0.8434
W1-R	2.044	7.504	2.230	2.416
W1-T	1.7344×10^{-6}	1.7643×10^{-5}	1.6316×10^{-6}	2.0842×10^{-6}
W1-P	0.2390	0.2286	0.2350	0.2377
